# Supplementary material for: A high-content endogenous GLUT4 trafficking assay reveals new aspects of adipocyte biology
Source: Life Sci Alliance. 2022 Oct 25;6(1):e202201585. doi: 10.26508/lsa.202201585 (PMC9595207; doi:10.26508/lsa.202201585)
Supplement: Supplementary file 1 [file LSA-2022-01585_TableS1.pdf]

Genes	log2FC_B	padj_B	minuslog10p	log2FC_I	padj_I	minuslog10p
Bcl9l.1	9.44841924	0.0353556	1.45154185	8.61918058	0.03889348	1.41012325
Clptm1	2.28775354	0.09178681	1.0372197	2.82897107	0.03889348	1.41012325
Gm14569	1.93404382	0.10555751	0.97651087	2.70709957	0.00799917	2.09695481
Hsd17b12	2.49866427	0.01905815	1.71991915	2.03834187	0.07801842	1.10780283
Jagn1	2.22100365	0.01043458	1.98152493	1.73261153	0.07839278	1.10572396
Pygo2	6.33230209	0.01743761	1.7585131	5.76687112	0.02057877	1.68658057
Rnf20	7.92484658	0.04672861	1.33041713	8.60919448	0.02315715	1.63531485
Tmem33	3.2227062	0.01817434	1.74054132	3.75831842	0.02956546	1.52921533
Ylpm1	9.16732951	0.00304907	2.51583293	9.56375568	0.00517327	2.28623478
